# Stromal Fibroblasts Drive Host Inflammatory Responses That Are Dependent on Chlamydia trachomatis Strain Type and Likely Influence Disease Outcomes

**DOI:** 10.1128/mBio.00225-19

**Published:** 2019-03-19

**Authors:** Amber Leah Jolly, Sameeha Rau, Anmol K. Chadha, Ekhlas Ahmed Abdulraheem, Deborah Dean

**Affiliations:** a Center for Immunobiology and Vaccine Development, UCSF Benioff Children's Hospital Oakland Research Institute, Oakland, California, USA; b Department of Bioengineering, University of California at Berkeley, Berkeley, California, USA; c Department of Medicine and Pediatrics, University of California at San Francisco, San Francisco, California, USA; Sequella, Inc.

**Keywords:** *Chlamydia trachomatis*, immune response, primary human epithelial cells, primary human stromal fibroblasts, sexually transmitted diseases, strain types, trachoma

## Abstract

Chlamydia trachomatis ocular strains cause a blinding disease known as trachoma. These strains rarely cause urogenital infections and are not found in the upper genital tract or rectum. Urogenital strains are responsible for a self-limited conjunctivitis and the sequelae of infertility, ectopic pregnancy, and hemorrhagic proctitis. However, the differential cellular responses that drive these clinically observed disease outcomes are not completely understood. Primary conjunctival, endocervical, and endometrial epithelial and stromal fibroblast cells, HeLa229 cells, and immortalized conjunctival epithelial (HCjE) cells were infected with the ocular A/Har-13 (A) and Ba/Apache-2 (Ba) strains and urogenital D/UW-3 (D) and E/Bour (E) strains. Infection rates, progeny production, and cytokine/chemokine secretion levels were evaluated in comparison with those in uninfected cells. All strain types infected all cell types with similar levels of efficacy and development. However, progeny production levels differed among primary cells: Ba produced significantly more progeny than E in endocervical and endometrial fibroblasts, while A progeny were less abundant than E progeny. C. trachomatis infection of primary epithelial cells elicited an increase in pro- and anti-inflammatory mediators compared to levels in uninfected cells, but there were no significant differences by strain type. In contrast, for primary fibroblasts, ocular strains elicited significant increases in the pro- and anti-inflammatory mediators macrophage inflammatory protein (MIP)-1β, thymus- and activation-regulated chemokine (TARC), interleukin (IL)-2, IL-12p70, and interferon gamma-induced protein 10 (IP-10) compared to levels in urogenital strains, while urogenital strains elicited a distinct and significant increase in the proinflammatory mediators IL-1α, IL-1β, IL-8, gamma interferon (IFN-γ), and granulocyte-macrophage colony-stimulating factor (GM-CSF). Our data indicate that primary fibroblasts, not epithelial cells, drive host inflammatory responses that are dependent on strain type and likely influence disease outcomes, establishing their importance as a novel model for studies of C. trachomatis disease pathogenesis.

## Abstract (precis)

Chlamydia trachomatis is a human pathogen and the leading cause of preventable blindness and sexually transmitted diseases in the world. Certain C. trachomatis strains cause ocular disease, while others cause upper genital tract pathology. However, little is known about the cellular or immunologic basis for these differences. Here, we compared the abilities of the strain types to infect, replicate, and initiate an immune response in primary human ocular and urogenital epithelial cells, as well as in fibroblasts from the underlying stroma. While there were no significant differences in infection rates or intracellular growth for any strain in any cell type, proinflammatory responses were driven not by the epithelial cells but by fibroblasts and were distinct between ocular and urogenital strains. Our findings suggest that primary fibroblasts are a novel and more appropriate model for studies of immune responses that will expand our understanding of the differential pathological disease outcomes caused by various C. trachomatis strain types.

## INTRODUCTION

Chlamydia trachomatis is an obligate intracellular bacterium that is tropic only for humans. C. trachomatis is an especially important public health threat, as it is the leading cause of preventable blindness (1, 2) and bacterial sexually transmitted infections in the world today (3, 4).

The organism has a biphasic developmental cycle (5) that consists of a dividing intracellular form, the reticulate body (RB), and a spore-like infectious and nonreplicating form, the elementary body (EB). C. trachomatis development begins when the EB infects the cell and morphologically changes into an RB within a phagocytic vacuole, called the inclusion. The inclusion is a protected intracellular compartment that allows the bacterium to parasitize the host for energy and nutrients and subvert intracellular detection.

C. trachomatis strains D/UW-3 (D) through K/UW-31/Cx (K) as well as lymphogranuloma venereum (LGV) strains cause lower urogenital tract infections that can lead to pelvic inflammatory disease (PID), chronic pelvic pain, infertility, and ectopic pregnancy (6). They can also cause proctitis. However, only the LGV strains can spread via lymphatics and cause diseases such as the inguinal syndrome and suppurating buboes (7, 8). All C. trachomatis strains can infect the ocular conjunctival mucosa, but strains A/Har-13 (A), B/TW-5/OT (B), Ba/Apache-2 (Ba), and C/TW-3/OT (C) typically are associated with the blinding ocular disease referred to as trachoma and rarely infect the urogenital tract (1, 9, 10). Importantly, some B and C strains are actually urogenital strains that have historically been misclassified (11–14). Non-LGV urogenital strains tend to cause a self-limited conjunctivitis, while LGV strains are especially virulent, constituting an ocular emergency to control infection (8, 15, 16).

The endocervix, endometrium, and conjunctiva are comprised of mucosal epithelium. C. trachomatis infects the single-layer columnar epithelial cells of the endocervix (reviewed in reference 17), the ciliated columnar epithelial cells of the endometrium (18), and the stratified squamous epithelial, stratified columnar epithelial and goblet cells of the conjunctiva (19, 20). The underlying resident fibroblasts in the stromal layer are subsequently infected. Following a complex series of events, many cells, including neutrophils, macrophages, monocytes, dendritic cells, polymorphonuclear cells, and T and B cells are recruited to these sites of infection (17, 19–26). Having a clearer understanding of how the resident cells that comprise the bulk of these healthy tissues respond to C. trachomatis infection is an essential first step in deciphering disease pathogenesis.

While C. trachomatis can infect a plethora of different cell types, most *in vitro* studies have focused heavily on human cervical carcinoma epithelioid HeLa229 cells (27, 28), human laryngeal carcinoma Hep-2 cells that are known to be contaminated with HeLa cells (29), and murine heteroploid fibroblast McCoy cells. Yet these cells differ in mediators of innate immunity, immune signaling pathways, and responses to Toll-like receptor agonists (30–32), making them less ideal for C. trachomatis studies.

Currently, there are 16 publications that evaluate one or at most two C. trachomatis strains separately in a single human primary cell type. These studies focused on a variety of subjects and examined the effects of antibiotics or hormones on C. trachomatis development, analyzed C. trachomatis-induced apoptotic pathways or persistence, or analyzed the secretion of the immune response marker IL-6 or IL-8 (18, 33–47). The limitation of these studies is that only C. trachomatis strains that are tropic for the respective primary cells were investigated. For example, primary human conjunctival epithelial cells were infected with ocular C. trachomatis strain B or C (33, 36), while primary human cervical and endometrial cells were infected with urogenital strains, such as E/Bour (E) and LGV/L2-434 (L2) (L_2_) (18, 34, 35, 39–41, 44–47).

*In vitro* studies that use primary cells are essential to advance our understanding of disease mechanisms, which can then be validated in appropriate animal models. While human studies are critically important, it is not possible to tease out the differential host responses to C. trachomatis infection in diverse cell types, especially at different tissue levels, such as the epithelial and stromal cell layers. The goals of our study were to compare the infection rates, levels of progeny production, and immune responses of C. trachomatis ocular and urogenital strains in a diversity of primary and immortalized human conjunctival and genital epithelial cells and stromal fibroblasts that are relevant for studying disease pathogenesis. Our data suggest that primary fibroblasts are a more appropriate model for studies of C. trachomatis tissue tropism and immunopathogenesis than other cell types. Specifically, stromal fibroblasts, not epithelial cells, drive the initial host inflammatory response, which is dependent on the infecting C. trachomatis strain type. This type of model provides the opportunity to expand our understanding of the differential pathological disease outcomes caused by various C. trachomatis strain types.

## RESULTS

### Ocular and urogenital strains of C. trachomatis infect immortalized and primary ocular and urogenital cells with similar efficacies.

Reference strains Ba/Apache-2 and E/Bour were used to infect HeLa 229 and immortalized human conjunctival epithelial (HCjE) cells, primary conjunctival epithelial (CjE) cells and stromal fibroblasts (CjS cells), primary endocervical epithelial (EcE) cells and stromal fibroblasts (EcS cells), and endometrial stromal fibroblasts (EmS cells). Cell composition and type were validated prior to our performing each assay using cytokeratin- and fibronectin-specific antibodies (see Fig. S1 in the supplemental material). Table S1 shows the origins of human primary cells by patient age and sex for CjE and CjS cells and by age for EcE, EcS, and EmS cells.

10.1128/mBio.00225-19.1 TABLE S1 Patient sources of primary cells and cell population characteristics used in designated figures. Download Table S1, PDF file, 0.1 MB. Copyright © 2019 Jolly et al. 2019 Jolly et al. This content is distributed under the terms of the Creative Commons Attribution 4.0 International license.

10.1128/mBio.00225-19.3 FIG S1 Primary cell composition and cell type validation. Each cell type was grown to 80% confluence on glass bottom 24-well plates prior to being fixed and stained (see Materials and Methods). The cells were labeled with squamous epithelial cell-specific cytokeratin 4 antibody (green), goblet cell-specific cytokeratin 7 antibody (red), columnar epithelial cell-specific cytokeratin 18 antibody (green), stromal cell-specific fibronectin antibody (cyan), and Hoesch dye (blue) for nuclear and bacterial DNA. Primary conjunctival epithelial (CjE) cell preparations had a range of 40 to 80% goblet cells and 20 to 60% squamous cells, depending on the dissection (see Materials and Methods). All stromal cells were verified to be free of epithelial cell contamination. Imaging was performed on a Nikon Eclipse Ti-E inverted microscope with an LED illumination system and a DS-Qi2 camera at 90× magnification. Download FIG S1, PDF file, 5.7 MB. Copyright © 2019 Jolly et al. 2019 Jolly et al. This content is distributed under the terms of the Creative Commons Attribution 4.0 International license.

**FIG 1:**
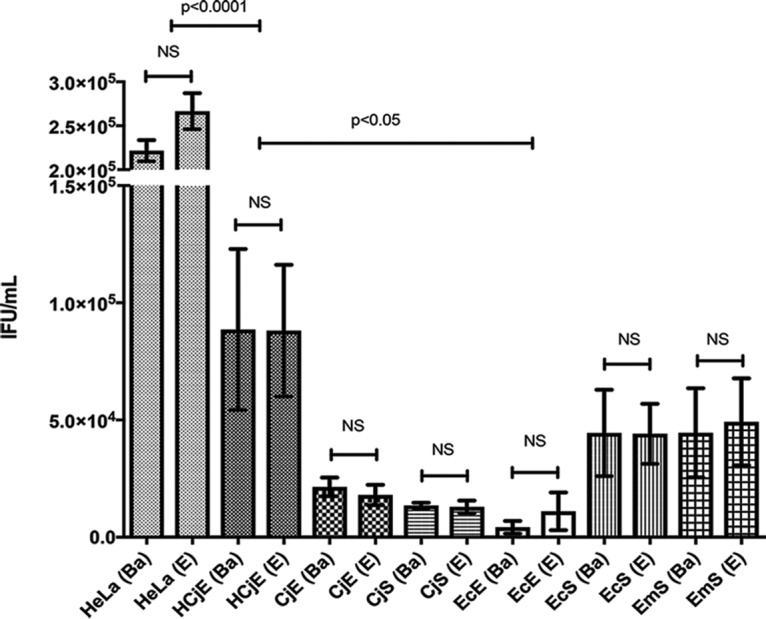
Ocular and urogenital strains of C. trachomatis infect immortalized and primary ocular and urogenital cells with similar efficacies. Immortalized HeLa 229 and human conjunctival epithelial (HCjE) cells and human primary conjunctival epithelial (CjE) cells, conjunctival stromal fibroblasts (CjS cells), endocervical epithelial (EcE) cells, endocervical stromal fibroblasts (EcS cells), and endometrial stromal fibroblasts (EmS cells) were infected at the same time with Ba/Apache-2 or E/Bour at an MOI of 1. At 48 hpi, the cells were fixed and stained, and the numbers of IFU per milliliter were determined (see Materials and Methods). The same medium was used for all infections. Data are presented as the sample means and standard errors of the means (SEM), with error bars reflecting three to four independent experiments for HeLa and HCjE cells and two for primary cells from four to five patients. Student's *t* test was used to determine whether there was a significant difference in infection efficacy between Ba and E strains for each cell type. NS, differences were not significant.

To examine the relative ability of each strain to be internalized and form inclusions by each cell type, cells were infected in the same medium and with the same stock of the respective C. trachomatis strain. We found that a multiplicity of infection (MOI) of 1 in HeLa cells corresponded to an MOI of <1 in other cell types, irrespective of the patient of origin of the cells. Both the Ba and E strains were able to infect and form inclusions in all seven cell types. Immortalized cells had the highest infection rates (Fig. 1). EcE cells had the most varied rates, and this was likely due to the abundance of mucus secreted by these cells, which was visible in the medium (unquantified observation). There were no significant differences when we compared primary cells to one another.

Infection rates also did not vary significantly when we compared primary cells isolated from multiple patients. Patient-to-patient variability is reflected in the standard deviation in Fig. 1. There were no strain-dependent differences in infection rate for the same cell type, irrespective of patient of origin, and the rates for both Ba and E were nearly identical in all seven cell types (Fig. 1).

### The developmental cycles for ocular and urogenital strains of C. trachomatis are similar in immortalized and primary cells.

**FIG 2:**
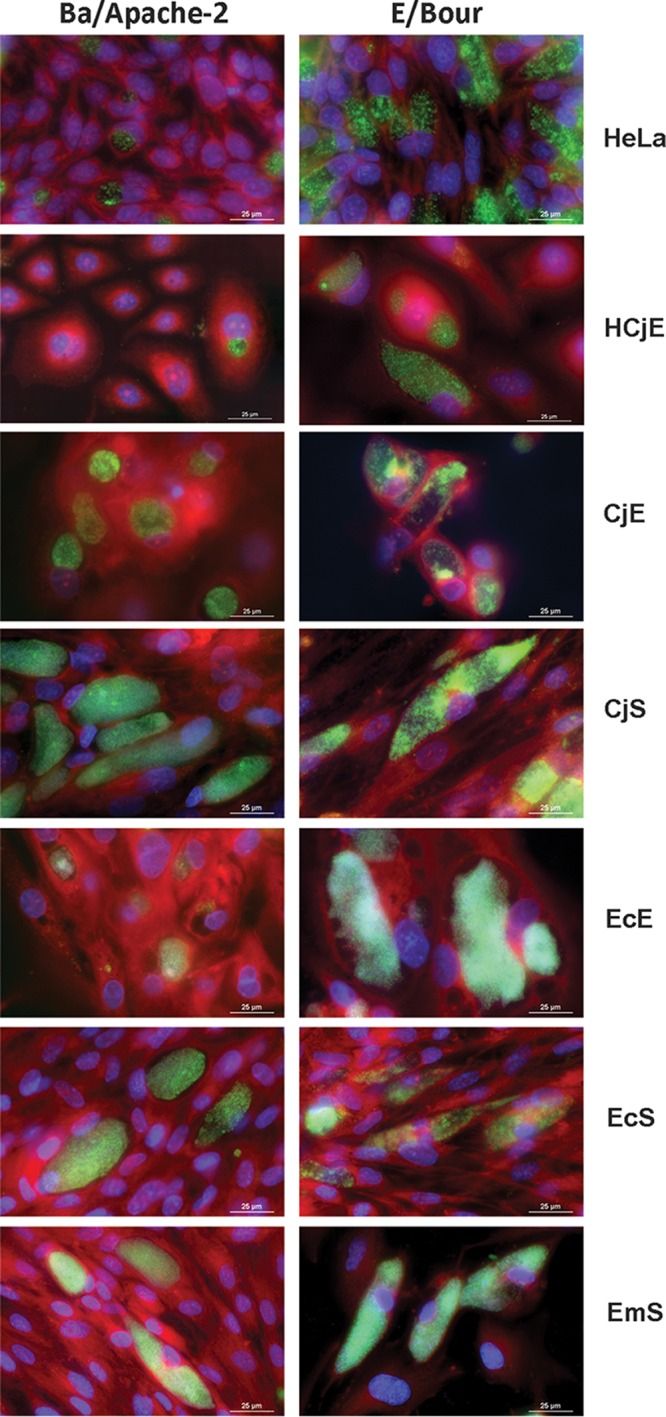
Representative images of inclusion formation by C. trachomatis reference strains Ba/Apache-2 and E/Bour. CjE, CjS, EcE, EcS, and EmS cells and immortalized HeLa 229 and HCjE cells were infected simultaneously with Ba/Apache-2 or E/Bour at an MOI of 1. The same medium was used for all infections. The cells were grown on 24-well glass-bottom plates, fixed and stained at 48 hpi, and imaged using a Nikon Eclipse Ti-E inverted microscope with an LED illumination system and a DS-Qi2 camera at ×90 magnification. Representative images are shown from more than three independent experiments.

Both the Ba and E strains are known to have an approximate 44- to 48-h developmental cycle in various cell lines (48). Similarly to what has been observed by others, strain E grew somewhat faster than Ba in HeLa cells, where inclusions were mature at 40 to 44 h postinfection (hpi). For all cell types, Ba and E inclusions were visible at 30 hpi, with a consistent increase in the inclusion area of approximately 2- to 5-fold until they reached their full size at 44 to 48 hpi (Fig. S2). The overall developmental cycle was independent of cell type or patient origin of the cells. Representative images of mature inclusions in each cell type for each strain at 44 to 48 hpi are shown in Fig. 2.

10.1128/mBio.00225-19.4 FIG S2 C. trachomatis inclusion areas at 30 and 48 hpi. HeLa229 and HCjE cells and primary CjE, CjS, EcE, EcS, and EmS cells were grown on tissue culture-treated plastic plates and infected with Ba/Apache-2 or E/Bour at an MOI of 1. The cells were fixed and stained at 30 or 48 hpi (see Materials and Methods). For determination of inclusion areas, images were acquired using a 40 by 1.5× air objective with an NA of 0.6 on a Nikon Eclipse Ti-E inverted microscope. Elements software was used to calculate inclusion areas expressed as square micrometers. Each filled or open circle represents one patient sample for the specified cell type. The horizontal line represents the mean inclusion area. Download FIG S2, PDF file, 1.6 MB. Copyright © 2019 Jolly et al. 2019 Jolly et al. This content is distributed under the terms of the Creative Commons Attribution 4.0 International license.

Ba and E inclusion areas revealed a biologically reproducible pattern at 44 to 48 hpi for replicate experiments in HeLa and HCjE cells and for CjE, CjS, EcE, EcS, and EmS cells from three different patients, although the area varied by the patient origin of the cell types (Fig. S3). E consistently formed significantly larger inclusion areas than Ba in epithelial cells. This pattern was not seen in stromal fibroblasts.

10.1128/mBio.00225-19.5 FIG S3 Variation in C. trachomatis mature inclusion area is dependent on patient of origin for each primary cell type. HeLa 229 and HCjE cells and primary CjE, CjS, EcE, EcS, and EmS cells were infected with Ba/Apache-2 or E/Bour at an MOI of 1 and fixed and stained at 48 hpi (see Materials and Methods). For determination of the inclusion areas, images were acquired using a 40 by 1.5× air objective with an NA of 0.6 on a Nikon Eclipse Ti-E inverted microscope. Elements software was used to calculate the inclusion areas, expressed as square micrometers. The results of three independent experiments for immortalized cells and cells from three patients for each primary cell type were compared. For CjE and EcE cell populations, inclusion areas were measured only for epithelial cells and not for any contaminating stromal cells, if present. NS, not significant. Download FIG S3, PDF file, 0.9 MB. Copyright © 2019 Jolly et al. 2019 Jolly et al. This content is distributed under the terms of the Creative Commons Attribution 4.0 International license.

### Levels of infectious progeny production by C. trachomatis ocular and urogenital strains differ among primary cell types.

We compared levels of infectious progeny production, expressed as inclusion-forming units (IFU) per milliliter, for strains Ba and E in each cell type using a standard reinfectivity assay that we previously described (52, 53). There were similar numbers of IFU per milliliter for both Ba and E in every cell type for the primary infection (Fig. S4). At 30 and 44 to 48 hpi, cells were lysed, and the same cells as in the primary infection were infected. We observed an increase in infectious progeny for all cell types between 30 and 44 to 48 hpi for both strains (data not shown). At 44 to 48 hpi, E consistently produced significantly more infectious progeny than Ba in HeLa and HCjE cells (Fig. S4) (*P* < 0.05). CjE, CjS, and EcE cells from different patients, independent of age and gender, produced similar numbers of progeny regardless of strain type. However, there was a significantly higher number of progeny produced by Ba than by E in primary EcS cells from every patient, independent of age and gender (Fig. S4) (*P* < 0.05), with a similar trend for EmS cells from some patients. Figure S4 shows the means of results from three to four independent experiments using immortalized cells and from experiments using primary cells from 3 to 6 patients.

10.1128/mBio.00225-19.6 FIG S4 Levels of infectious progeny production by C. trachomatis ocular and urogenital strains differ among cell types. HeLa 229 and HCjE cells and primary CjE, CjS, EcE, EcS, and EmS cells were infected with Ba/Apache-2 or E/Bour at an MOI of 1. At 48 hpi, the cultures were serially diluted onto the same cell types from the same patients as the primary infection. Cells were fixed and stained at 48 hpi, and the number of IFU per milliliter was determined (see Materials and Methods). The values for the primary infection represent the mean and standard deviation for three to four independent experiments for immortalized cells and for three to six patients per cell-type for primary cells. The values for the re-infection represent three to four independent experiments/patients for each cell type. Download FIG S4, PDF file, 1.8 MB. Copyright © 2019 Jolly et al. 2019 Jolly et al. This content is distributed under the terms of the Creative Commons Attribution 4.0 International license.

**FIG 3:**
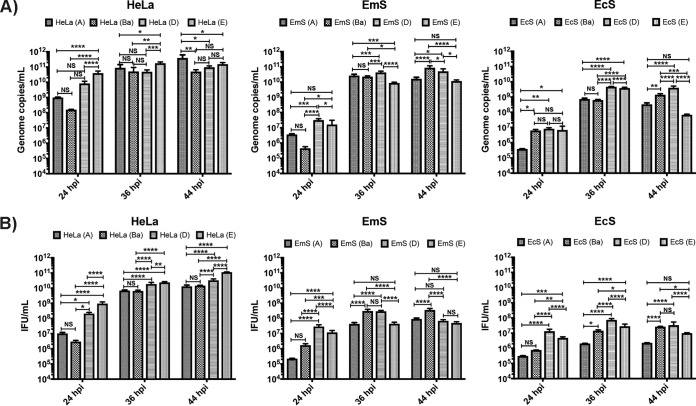
Infectious progeny production by C. trachomatis strains A/Har-13, Ba/Apache-2, D/UW-3, and E/Bour in HeLa, EcS, and EmS cells. HeLa 229 cells and primary EcS and EmS cells from the same patient were infected with an ocular or urogenital C. trachomatis strain. At 24, 36, and 44 hpi, the cultures were serially diluted onto the same cell types from the same patients as the primary infection. (A) C. trachomatis genome copy numbers by strain and cell type at designated time points. Genomic DNA was extracted from each culture, and qPCR was performed to determine the genome copy number for each reinfectivity assay (see Materials and Methods). (B) Numbers of IFU per milliliter by strain and cell type at designated time points. Numbers of IFU per milliliter were determined for each reinfectivity assay (see Materials and Methods). A one-way ANOVA was performed at each time point (with a Fishers LSD posttest). *, *P* < 0.05; **, *P* < 0.01; ***, *P* < 0.005; ****, *P* < 0.001; NS, not significant. The data reflect three independent experiments for HeLa cells and for primary cells from 3 to 6 patients.

To determine whether the differences in progeny production in EcS and EmS cells were due to a difference in ocular versus urogenital strain growth and differentiation of RBs to EBs, we performed another infectivity assay by simultaneously infecting HeLa cells, as well as EcS and EmS cells from the same patient, with the reference ocular strains A/Har-13 and Ba/Apache-2 and the reference urogenital strains D/UW-3 and E/Bour at an MOI of 1. The infectious strains were passaged at 24, 36, and 44 hpi to an uninfected monolayer of the same cells. Genomic DNA copy number was determined by quantitative PCR (qPCR) (Fig. 3A), as were numbers of IFU per milliliter (Fig. 3B) for each time point. In HeLa cells, urogenital strains produced a higher number of infectious progeny than ocular strains throughout development, as has been documented previously (48). Consistent with our prior findings for EcS cells, Ba produced significantly more progeny than E at 44 hpi (*P* < 0.05), which was corroborated by genome copy number (*P* < 0.005). These findings were similar for EmS cells (*P* < 0.001 for both). The progeny production of A was significantly lower than for Ba in EcS and EmS cells at 36 and 44 hpi. D was similar to Ba only at 44 hpi in EcS cells.

### Stromal fibroblasts produce a distinct immune response, depending on infection with ocular versus urogenital strain types.

**FIG 4:**
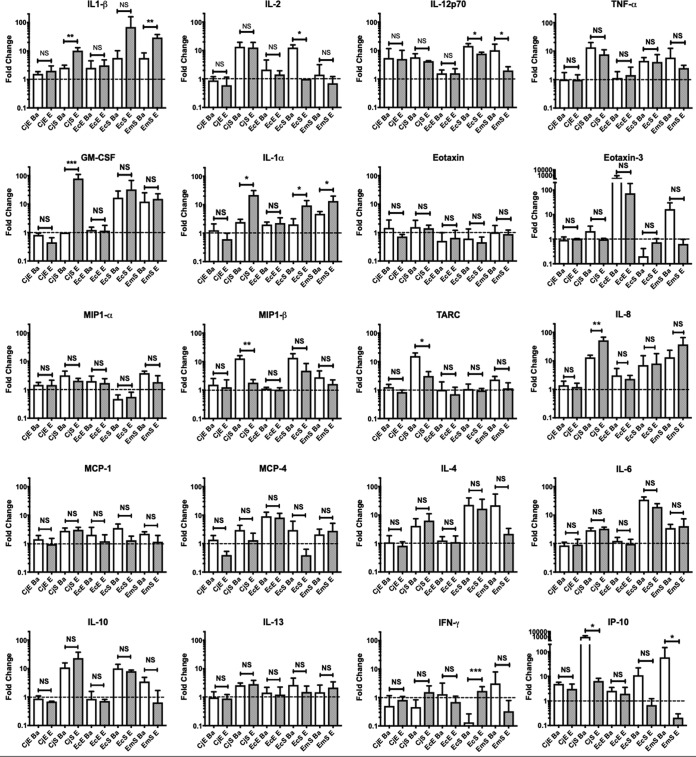
Cytokine and chemokine secretion levels vary by cell type following infection with C. trachomatis ocular and urogenital strains. Immortalized HeLa 229 and HCjE cells and primary CjE, CjS, EcE, EcS, and EmS cells were infected with Ba/Apache-2 or E/Bour at an MOI of 1 or mock infected. Supernatants were collected at 48 hpi and analyzed using the Meso Scale Discovery human cytokine/chemokine V-PLEX arrays for 20 analytes (see Materials and Methods). The data were analyzed by compiling the fold changes of secreted analyte versus uninfected cells. Student's *t* test was performed on the log-transformed fold change data. *, *P* < 0.05; **, *P* < 0.01; ***, *P* < 0.005; and ****, *P* < 0.001; NS, not significant. The data reflect three independent experiments for HeLa cells and for primary cells from 4 patients.

HeLa, HCjE, CjE, CjS, EcE, EcS, and EmS cells were mock infected or infected with Ba or E. Primary cells were from four different patients. Supernatants were collected at 44 to 48 hpi and subjected to meso-scale detection (MSD) quantification of 29 cytokines and chemokines. Reproducible patterns of analytes were apparent for the same cell type from multiple patients, after normalization to the basal secretion rates of mock-infected cells (Fig. 4); 20 analytes were up- or downregulated by C. trachomatis infection compared to their levels in mock-infected controls (Table S2).

10.1128/mBio.00225-19.2 TABLE S2 Analytes that were up- or down-regulated in response to C. trachomatis compared to levels in mock-infected primary cells (data were taken from Fig. 4). Download Table S2, PDF file, 0.1 MB. Copyright © 2019 Jolly et al. 2019 Jolly et al. This content is distributed under the terms of the Creative Commons Attribution 4.0 International license.

**FIG 5:**
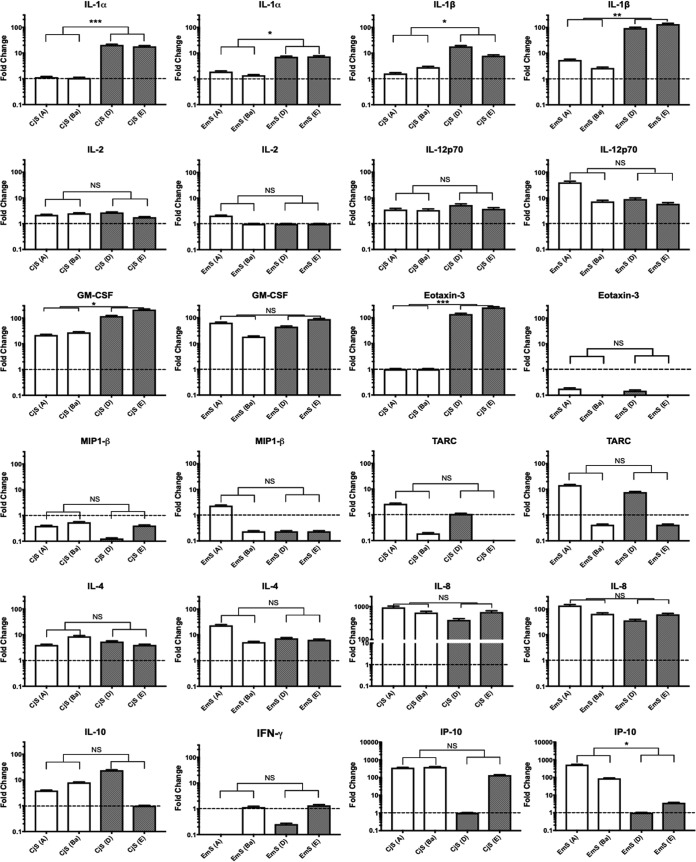
C. trachomatis ocular and urogenital strains elicit distinct patterns of cytokines and chemokines in CjS cells and EmS cells. Primary CjS and EmS cells from the same patient were infected with A/Har-13, Ba/Apache-2, D/UW-3, and E/Bour at an MOI of 1 or mock infected for 48 h. Two independent experiments were performed. The supernatants were analyzed using the Meso Scale Discovery human cytokine/chemokine V-PLEX arrays for 20 analytes (see Materials and Methods). Student's *t* test was performed on the log-transformed fold change data. *, *P* < 0.05; **, *P* < 0.01; ***, *P* < 0.005; ****, *P* < 0.001; NS, not significant.

There were no significant C. trachomatis strain-dependent differences in cytokine or chemokine secretion following infection of immortalized or primary epithelial cells (Fig. 4). However, CjS, EcS, and EmS cells showed significant differences when we compared Ba and E infections for 10 analytes, as shown in Table 1 (Fig. 4). To determine whether these findings were consistent for other ocular and urogenital strains, CjS and EmS cells were simultaneously infected with the reference strains A/Har-13, Ba/Apache-2, D/UW-3, and E/Bour for 44 to 48 h at an MOI of 1. This assay confirmed that, in CjS cells, urogenital strains significantly enhanced the secretion of IL-1α, IL-1β, granulocyte-macrophage colony-stimulating factor (GM-CSF), and eotaxin-3 compared to the levels of secretion produced by ocular strains (Fig. 5). In EmS cells, urogenital strains similarly significantly enhanced interleukin (IL)-1α and IL-1β secretion, while ocular strains enhanced interferon gamma-induced protein 10 (IP-10) secretion (Fig. 5).

**TABLE 1:** Comparison of primary fibroblast cell analyte levels for C. trachomatis ocular Ba and urogenital E infections^*a*^

Cytokine or chemokine	Fold change in analyte level in CjS cells infected with:		*P* value	Fold change in analyte level in EcS cells infected with:		*P* value	Fold change in analyte level in EmS cells infected with:		*P* value
	Ba	E		Ba	E		Ba	E	
Proinflammatory cytokines									
IL-1α	2.45 ± 1.05	21.9 ± 16.24	***0.0132***	2.03 ± 1.17	9.4 ± 4.54	***0.0137***	4.77 ± 0.97	13.49 ± 6.51	***0.0197***
IL-1β	2.58 ± 1.19	10.22 ± 5.66	***0.0077***	5.68 ± 4.57	69.39 ± 89.67	0.0570	5.65 ± 2.91	29.68 ± 8.1	***0.0030***
GM-CSF	1 ± 0	78.4 ± 54.16	***0.0030***	16.68 ± 11.94	32.62 ± 34.67	0.6887	12.19 ± 12.66	15.12 ± 7.83	0.4779
IFN-γ	0.4602 ± 0.7308	1.542 ± 2.037	0.7248	0.1339 ± 0.1313	1.696 ± 0.7026	***0.004***	3.113 ± 4.783	0.3289 ± 0.4524	0.4553
Regulatory T cells/Th2 cytokines									
IL-2	13.54 ± 10.23	12.73 ± 11.33	0.8005	12.67 ± 3.083	1 ± 0	***<0.0001***	1.49 ± 1.77	0.71 ± 0.51	0.7713
TARC (CCL17)	15.59 ± 7.76	3.12 ± 2.28	***0.0407***	1.09 ± 0.53	0.96 ± 0.17	0.3411	2.34 ± 0.68	1.15 ± 0.65	0.5980
Chemokines									
IP-10 (CXCL10)	1,036 ± 1,239	6.45 ± 3.21	***0.0101***	11.13 ± 10.93	0.68 ± 0.56	0.0509	59.17 ± 89.17	0.21 ± 0.09	***0.0321***
IL-12p70	5.78 ± 3.39	4.23 ± 0.28	0.6334	14.44 ± 3.02	7.79 ± 0.93	***0.0122***	10.25 ± 6.49	1.99 ± 0.71	***0.0411***
MIP-1β	12.83 ± 5.68	1.82 ± 0.88	***0.0099***	13.57 ± 5.36	4.76 ± 3.79	0.1209	2.81 ± 1.89	1.64 ± 0.63	0.5105
IL-8 (CXCL8)	13.25 ± 5.06	52.54 ± 26.65	***0.0100***	7.09 ± 8.07	8.08 ± 9.93	0.9855	13.36 ± 10.17	37.7 ± 28.29	0.1150

a The cytokines and chemokines with strain-dependent differences are shown. Data are the same as in Fig. 4 and represent fold changes (with averages and standard deviations for four patients) from levels in uninfected cells. Underlining indicates a trend in the data; boldface italics indicate statistically significant *P* values.

### Cytokine and chemokine secretion varies by MOI for C. trachomatis ocular and urogenital strains.

We evaluated the impact of Ba and E strain MOIs of 1 and 10 on cytokine and chemokine secretion using CjE and CjS cells from a representative male patient and EcE, EcS, and EmS cells from a representative female patient. Using the same cells from a patient allowed us to compare within-host immune responses for simultaneous infections with Ba and E (Fig. S5; Table 2). Consistent with the above findings, CjE and EcE cells were less immunoreactive than CjS, EcS, and EmS cells. However, at an MOI of 10 in CjE cells, the proinflammatory mediators eotaxin, thymus- and activation-regulated chemokine (TARC), monocyte chemoattractant protein-1 (MCP-1), gamma interferon (IFN-γ), and IP-10 were significantly upregulated by Ba compared to levels produced by E, while E enhanced the secretion of anti-inflammatory cytokine IL-10 compared to that produced by Ba. At an MOI of 10 for EcE cells, only anti-inflammatory IL-10 (i.e., E produced more than Ba) and proinflammatory eotaxin-3 (i.e., Ba produced more than E) were significantly upregulated.

**TABLE 2:**
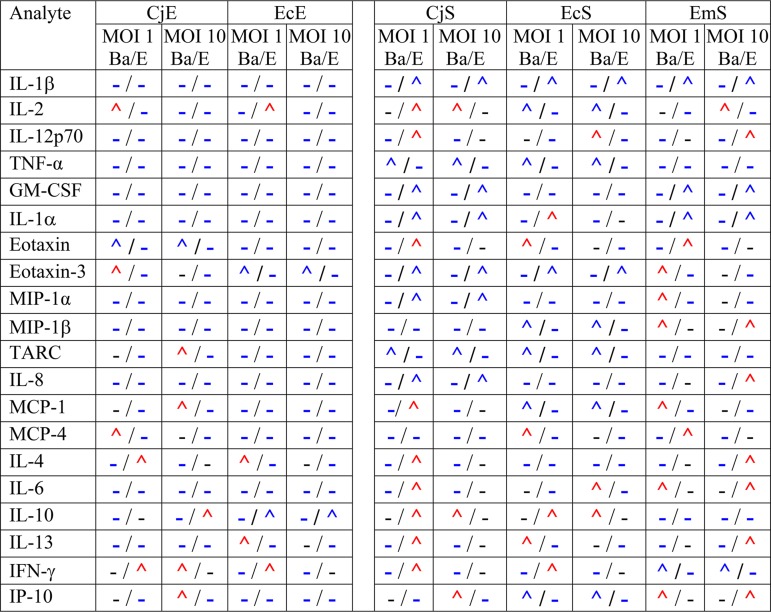
Comparison of significant differences in cytokine and chemokine levels for C. trachomatis reference strains Ba/Apache-2 and E/Bour^*a*^

a Cells of each primary cell type were infected with Ba or E at MOIs of 1 or 10 (data are taken from Fig. S5 in the supplemental material). Symbols before a slash relate to strain Ba; symbols after a slash relate to strain E. Blue and red carets represent up-regulation for one strain versus the other strain. Hyphens represent no significant up-regulation. Red carets indicate that there was a difference in analyte results for the cell type infected with strain Ba or E at an MOI of 1 compared to an MOI of 10; blue carets and hyphens indicate a similarity in results in the cell type infected at MOIs of 1 and 10. Patient and cell population information can be found in Table S1.

10.1128/mBio.00225-19.7 FIG S5 Cytokine and chemokine secretion varies depending on the MOI for C. trachomatis ocular and urogenital strains. Immortalized HeLa229 and HCjE cells and primary CjE, CjS, EcE, EcS, and EmS cells were infected with Ba/Apache-2 or E/Bour at an MOI of 1 or 10 or mock infected. The supernatants were collected at 48 hpi and analyzed using the Meso Scale Discovery human cytokine/chemokine V-PLEX arrays for 20 analytes (see Materials and Methods). The dotted line indicates the lower limit of detection (LLOD) for that analyte, based on the standard curve. To determine whether there was a significant increase in secretion between an MOI of 1 and an MOI of 10, a threshold for fold change was set at ±2; *, indicates a >2-fold increase (see Materials and Methods). The data reflect the results of three independent experiments using immortalized cells and for CjE and CjS cells from a representative male patient and for EcE, EcS, and EmS cells from a representative female patient. Download FIG S5, PDF file, 2.1 MB. Copyright © 2019 Jolly et al. 2019 Jolly et al. This content is distributed under the terms of the Creative Commons Attribution 4.0 International license.

As with the multiple-patient data (Table 1; Fig. 4), Ba and E upregulated many more cytokines and chemokines in fibroblasts than in other cell types (Fig. S5; Table 2). In CjS cells, the majority of mediators were significantly upregulated by E compared to levels of secretion produced by Ba and were proinflammatory primarily at an MOI of 1 and less frequently at an MOI of 10; Ba elicited significantly higher levels of IL-2, tumor necrosis factor alpha (TNF-α), TARC, IL-10, and IP-10 than E but only at an MOI of 10, except with TNF-α. For EcS cells, the majority of significant strain-dependent differences occurred at an MOI of 1, and often the same strain showed significantly upregulated mediators at an MOI of 10. The findings for EmS cells were less consistent (Fig. S5; Table 2).

## DISCUSSION

The physiologies of the cervix, endometrium, and conjunctiva are distinct, which has implications for the pathogenesis of disease. Additionally, while ocular and urogenital strain types can infect similar tissues in the human host, neither has been documented to cause severe pathology in nontropic tissue. The basis of the present study was, therefore, to explore the infectivity, progeny production, and immune responses of strains in primary human ocular and urogenital epithelial cells and stromal fibroblasts to those of C. trachomatis eye- and urogenital-tissue-tropic strains to expand our understanding of the roles that these cells and strain types may play in clinically observed differential disease outcomes. This type of study would be impossible to conduct in human populations.

C. trachomatis infects many cell types (49, 51), and therefore it was not surprising that ocular Ba and urogenital E strains had similar infection efficacies for all cell types in our study, although the rates of infection were highest for HeLa and HCjE cells. This latter finding was likely due to the laboratory adaptation of C. trachomatis reference strains to these cell lines. What was surprising, however, was that there were no strain-dependent differences in infection rate or developmental cycle regardless of whether the primary cells were from ocular or genital tissue. These data were consistent across primary cells from different patients.

The inclusion areas were consistently larger in epithelial cells for strain E than for strain Ba. Larger inclusion sizes are often equated with higher numbers of infectious progeny, as we and others have shown for immortalized cells (48, 52, 53). However, in primary EcE and CjE cells, levels of infectious progeny production were similar and independent of the variation in inclusion size, even with similar percentages of infection in the primary infection. This counterintuitive finding is supported by a recent report that showed that C. trachomatis replication does not necessarily correlate with inclusion size, even in immortalized cells (50).

Surprisingly, Ba produced significantly more infectious progeny in both EcS and EmS cells than E (Fig. S4). However, this pattern did not hold for strain A or D (Fig. 3) (D was similar to Ba). Prior research has identified that D can form recombinants with both ocular (i.e., Ba/D) (12, 54) and urogenital (i.e., Ba/D and D/L_2_) strains (55, 56), indicating that the intracellular adaptability of D may facilitate these natural transformation events. Yet, the fact that both ocular and urogenital strains produce progeny equally well in primary ocular but not genital cells suggests that the exposure to different C. trachomatis strains is not the determining factor in disease outcomes but that the innate immune response of the cell type is what drives disease. On the other hand, in the urogenital tract, disease progression may be modulated by both strain-dependent intracellular progeny production in specific cell types and strain-induced immune responses.

Studies using immortalized and primary genital epithelial cells support the hypothesis that the inflammatory response to C. trachomatis is initiated and perpetuated by epithelial cells (39, 57–61). The female reproductive tract is particularly susceptible to C. trachomatis infection of the columnar epithelium of the endocervix (62), and urogenital strains such as D and L_2_ have been shown to induce the production of IL-1α, IL-6, TNF-α, IL-10, IL-12, and GM-CSF (reviewed in reference 17), contributing to an adverse inflammatory response (reviewed in reference 63). We confirmed that C. trachomatis infection of EcE cells elicited similar responses as above compared to uninfected cells; these responses were consistent across cells from multiple patients, but responses were low. However, we found novel evidence that IL-1β, IL-2 eotaxin-3, IL-8, MCP-1, and IP-10 were also upregulated in these cells. When we compared Ba and E infections for cytokine and chemokine production in this cell type, there were no significant differences, except at an MOI of 10 (see below).

While the basal level of cytokine and chemokine secretion was higher for EcE cells than for EcS and EmS cells, infection-induced immune mediator levels were notably higher in EcS and EmS cells than in EcE cells. This may reflect the fact that epithelial cells already secrete maximal or nearly maximal amounts of many of the mediators analyzed here. However, while infected and uninfected epithelial cells are more readily sloughed, fibroblasts are long-term residents of the tissue and provide a relatively protective niche for maintenance of C. trachomatis infection. They are responsible for regulating tissue integrity through secretion of extracellular matrix, but more importantly, they control the transition from an acute and resolving form of inflammation to chronic inflammation, primarily via regulating chemotaxis (64–66).

In EcS and EmS cells, C. trachomatis induced upregulation of both pro- and anti-inflammatory responses compared to levels in uninfected cells. However, the response differed by C. trachomatis strain type: compared to ocular strains, urogenital strains induced significantly higher levels of the proinflammatory cytokines IL-1α, IL-1β, and IFN-γ, while the ocular strains elicited significantly higher levels of pro- and anti-inflammatory mediators (e.g., IL-2, IL-12p70, and IP-10, with a trend for IL-10 [*P* = 0.08] and eotaxin-3 [*P* = 0.0528]) (Table 1; Fig. 4 and 5). When we evaluated responses to an MOI of 10, the patterns for both the ocular and urogenital strains were similar to the patterns produced with an MOI of 1 in EcS cells, with additional differences in EmS cells (Table 2).

Conjunctival inflammation involves many cell types. The clinical presentation of trachoma is characterized by the formation of follicles (19–21, 25, 67) in the stroma that can resolve on their own or progress to severe inflammation and subsequent irreversible scar formation (68–70), with replacement of the stroma by type V and type VI collagen (22). Immune cells, including monocytes, lymphocytes, and macrophages, are recruited into the tissue (20, 21, 25, 67). While histology shows an intact epithelium lying over the follicles (19), chronic trachoma is known to damage the epithelium, in particular, goblet cells (71, 72).

Conjunctival epithelial cells are naturally sloughed approximately every 7 to 8 days (73–75) and replaced by epithelial stem cells located in the fornix and bulbar conjunctiva (73, 76, 77). CjS cells, on the other hand, like all fibroblasts, are found in low numbers within connective tissue, usually have low metabolic activity, and are responsible for the secretion of extracellular matrix, the major constituents of the connective tissue (i.e., fibronectin, collagen, etc.). Moreover, fibroblasts are considered to be the central player in the formation of scar tissue in the anterior chamber of the eye (reviewed in reference 78).

Both urogenital and trachoma strains can cause follicles, but only the ocular strains cause scarring of the upper and lower palpebral conjunctivae (79–81). In studies of immune responses in trachoma patients, various cytokines and chemokines have been verified to be upregulated by using antibody-based assays that detect these proteins in conjunctival swabs or sponges, sampling that does not reach the stroma. However, the cells and secretions that are obtained may be influenced by passive diffusion of secreted proteins or cell-cell signaling mechanisms from the stroma.

Many more immune mediators have been implicated at the transcriptional level but have not been validated by testing for secreted proteins. The response noted previously for individuals with trachomatous inflammation, follicular (TF), and trachomatous inflammation, intense (TI), included upregulation of IL-1α, IL-1β, IL-6, IL-8, IL-10, IL-15, CXCL9 (MIG), TNF-α, transforming growth factor β2 (TGF-β2), MCP-1, and eotaxin (20, 57, 58, 82–88) compared to levels in patients without TF or TI. TF reflects acute inflammation that can resolve, while TI can lead to scar formation and progressive disease (1). We found upregulation of a number of these cytokines and chemokines in CjE cells compared to their levels in uninfected cells, and these levels did not differ significantly by strain type (Fig. 4), except at an MOI of 10, at which Ba induced significantly higher levels of proinflammatory cytokines and chemokines (Fig. S5; Table 2), suggesting that load may play a role in disease pathogenesis.

Compared to uninfected cells, CjS cells elicited a more robust response than CjE cells and showed additional differences in immune responses by strain type: Ba elicited significantly higher levels of MIP-1β, TARC, and IP-10 than E, while E elicited distinctly different and significantly higher levels of IL-1α, IL-1β, GM-CSF, and IL-8 (Table 1). These trends held for A and D, except with IL-8 levels. At an MOI of 10, the responses for Ba and E were similar to those at an MOI of 1, except that Ba additionally induced significantly higher levels of IL-2, IL-10, and IP-10, reflecting both a pro- and anti-inflammatory response (Fig. S5; Table 2).

Previous studies in areas of trachoma endemicity have shown single infections with C. trachomatis urogenital strains as well as a much lower prevalence of coinfections of ocular and urogenital strains associated with TF and/or TI, suggesting that urogenital strains may contribute to disease (89, 90). Others have reported trachoma-like disease, with a single urogenital strain being reported among individuals residing in countries where trachoma is not endemic, such as Denmark and the United States (91, 92). These findings are supported by studies of nonhuman-primate models of trachoma where a urogenital strain, such as E, elicited severe disease similar to that of trachoma (93–95). Infectious load may play a role here (1), especially since we observed additional upregulation of inflammatory cytokines and chemokines with an MOI of 10. The ocular mucosa is constantly bathed in tears, which help remove and disperse EBs. Additionally, the mucosa contains gel-forming mucins that trap bacteria, preventing their binding to the ocular surface (96). This can result in movement of pathogens into the nasolacrimal duct during blinking (97). In endocervical C. trachomatis infections, a purulent discharge develops that moves antegrade into the vagina and sometimes retrograde into the endometrium develops. While some mucin is produced, it is not as efficient as tears in cleansing the area or dispersing the pathogen. It is therefore likely that the load of an ocular strain transmitted from one conjunctiva to another or to the urogenital tract is low but that the load of a urogenital strain transmitted to the conjunctiva is high. Following transmission, the load may change with associated resolution of infection or disease progression. Additional studies are needed to further elucidate the immune mediators in relation to infectious load in both primary epithelial cells and stromal fibroblasts.

The finding that urogenital strains elicit a proinflammatory response (e.g., IL-1α, IL-1β, GM-CSF, and IL-8 [for E]) in the conjunctiva distinct from that of ocular strains (Table 1; Table 2) suggests that the pathogenic mechanism of disease may be unique. Neutrophil recruitment plays an essential role in the initial clearing of a C. trachomatis infection (98–101), which is likely mediated in part by the secretion of IL-8 (reviewed in reference 102). IL-8 was enhanced 10- to 100-fold in all primary stromal fibroblasts following C. trachomatis infection compared to levels in uninfected primary cells (Fig. 4). In the ocular surface, neutrophil recruitment is an essential early innate immune response, and the successful resolution of an initial inflammatory response involves the phagocytosis of spent neutrophils by recruited macrophages (reviewed in reference 103). In addition, given the well-established role of GM-CSF in recruiting monocytes to sites of infection and stimulating the growth and differentiation of myelomonocytic lineage cells (reviewed in references 104 and 105), it is possible that this recruitment may help to resolve the conjunctival inflammatory response to urogenital strains, but that macrophage recruitment and activation are less effective in responding to ocular strains. This may in part contribute to the severe inflammation seen with repeated infection with ocular strains that appears to drive the development of scarring pathology in trachoma (2, 70, 90). Further studies that involve repeat infection of primary fibroblasts with the same and different C. trachomatis strain types are required.

The resolution of the inflammatory response in the urogenital tract, as in the eye, is likely to also require a balance between neutrophil and macrophage populations. Lijek et al. (106) compared the effects of transcervical inoculation of D with L_2_ in the murine genital tract model. They found that D uniquely induced an enhanced persistent neutrophil and monocyte inflammatory infiltrate of the uterine lumen and oviducts that resembled PID and lasted weeks, beyond when C. trachomatis organisms would be detected. The inflammation from L_2_, in contrast, was mild and transient. These findings may in part be due to the fact that L_2_ lacks the C. trachomatis toxin (107) that allows L_2_ dissemination via lymphatics to regional lymph nodes but, when the toxin is present, is known to increase virulence and toxicity at mucosal sites of infection (56). Furthermore, in the urogenital tract, T_H_2 cell recruitment is associated with prevention of inflammation, while T_H_1 and T_H_17 recruitment clears bacterial infection but results in devastating pathology (61, 108–110). In the present study, we found that T cell-regulating cytokines and chemokines, including IL-2 (which induces T cell proliferation), IL-12p70 (which induces CD4 T-cell differentiation into T_H_1-like cells), and IP-10 (which recruits activated T cells, especially T_H_1 cells), were upregulated by ocular strains in the urogenital fibroblasts, suggesting that these strains might cause progressive pathology. The details of the effects of these T cell-regulating cytokines and chemokines on disease pathology will be an important topic for future studies.

While the innate immune response to C. trachomatis infection involves many cells and a complex orchestration of cytokine and chemokine signaling, in addition to orchestration of lipid mediators of inflammation, among others, this study indicates that stromal fibroblasts drive the initial acute host inflammatory response and consequently may contribute to disease outcome. In addition, this response is dependent on the infecting C. trachomatis strain type. Our findings provide a novel step in understanding the differential immune responses of healthy human tissue to C. trachomatis strain types and form the basis for evaluating the downstream effects of these immune responses on cell migration, inflammation, pathology, and disease in future studies.

## MATERIALS AND METHODS

### C. trachomatis strains and infections.

C. trachomatis reference strains A/Har-13, Ba/Apache-2, D/UW-3, and E/Bour were propagated in HeLa cells, purified by density gradient centrifugation, and verified by *ompA* genotyping, and their titers in HeLa 229 cells were determined for numbers of IFU per milliliter and the MOI as previously described (56, 111–113). An MOI of 1 or 10 was used as indicated above. Cells were infected at 80% confluence in 24-well tissue culture-treated plates (Greiner Bio-One, Monroe, NC) or shell vials (E&K Scientific, Santa Clara, CA) in a 50:50 mix of RPMI 1640 (Lonza, Allendale, New Jersey) and KGM (Lonza) media without antibiotics at 37°C in 5% CO_2_. RPMI 1640 without l-glutamine (Lonza) was supplemented with 10% fetal bovine serum (FBS; Lonza), 2 mM l-glutamine (Lonza), 10 mM HEPES (Lonza), 1 mM sodium pyruvate (ThermoFisher, Waltham, MA), and a nonessential amino acid mixture (Lonza). To directly compare ocular and urogenital strain infectivities, cells were simultaneously infected with an MOI of 1.

### Immortalized and primary cell preparations.

HeLa229 cells were cultured in DMEM (Lonza) supplemented with 10% FBS (Lonza). HCjE cells were cultured in keratinocyte serum-free medium (ThermoFisher) supplemented with CaCl_2_ (Sigma, St. Louis, MO), 1.25 μg human recombinant epidermal growth factor (Thermo Fisher), and 0.2 ng/ml bovine pituitary extract (ThermoFisher). HeLa and HCjE cells were confirmed free of *Mycoplasma* contamination using the ATCC Universal mycoplasma detection kit (ATCC 30-1012K). EcE, EcS, and EmS cells were prepared from biopsied cancer-free hysterectomy tissue provided the same day as deidentified specimens from San Francisco Bay area hospitals. CjE and CjS cells were prepared from cancer-free tissue preserved in Optisol (Chiron Ophthalmics, Irvine, CA) and received deidentified (except for sex, age, and cause of death) from Saving Sight (Kansas City, MO) within 48 h of death. All clinical specimens were considered not human subject research by the Institutional Review Board of the UCSF Benioff Children’s Hospital, Oakland Research Institute.

Tissue dissections were performed in 24-well tissue culture dishes (E&K Scientific) by allowing epithelial cells to migrate off tissue plugs in RPMI 1640 medium before the medium was changed to KGM medium after 3 days. Migrated epithelial cells were harvested from days 7 to 10 by treating them with Versene (ThermoFisher) and TrpLE (ThermoFisher), with an approximate survival rate of 30%. These cells were used at passage 1, as they do not survive freezing or repeated passaging. Tissue plugs were then moved to fresh tissue culture dishes, and fibroblasts were collected 1 to 2 weeks later and used at passages 1 to 5.

### Characterization of cell types.

For cell composition characterization, immunofluorescence was performed using mouse monoclonal cytokeratin 4 antibody Ab9004 (Abcam, Cambridge, MA), specific for stratified squamous epithelial cells at a1:20 dilution, and rabbit cytokeratin 7 antibody Ab181598 (Santa Cruz Biosciences, Santa Cruz, CA), specific for goblet cells at a dilution of 1:200. Cervical cell composition was determined using the cytokeratin 7 antibody and mouse cytokeratin 18 antibody MAB3404 (EMD Millipore, Temecula, CA), specific for columnar epithelial cells at a 1:100 dilution. Sheep anti-human fibronectin antibody AF1918 was used to detect fibroblasts (R&D Systems) at 1:300. Secondary Alexa Fluor 488 goat anti-rabbit (A11008; Thermo Fisher), Alexa Fluor 568 goat anti-mouse (A11004; ThermoFisher), and Alexa Fluor 647 donkey anti-sheep A21448 (ThermoFisher) antibodies were diluted 1:500.

### Microscopy.

Imaging was performed on a Nikon Eclipse Ti-E inverted microscope with an LED illumination system and a DS-Qi2 camera. For quantitation of C. trachomatis inclusion areas, images were acquired using a 40 by 1.5× air objective with a numerical aperture (NA) of 0.6. Elements software was used to calculate inclusion areas using 50 infected cells for 10 different fields. Representative high-resolution Ba and E inclusion images were captured using a 60 by 1.5× oil objective with an NA of 1.4 after methanol fixation and staining with Pathfinder (*Chlamydia* confirmation system; Bio-Rad, Hercules, CA) according to the manufacturer’s instructions. Hoechst dye (Sigma) was used to stain nuclear and chlamydial DNA. Approximately 5,000 cells were imaged to determine percent infection based on numbers of IFU and also for numbers of IFU/ml, as we described previously (52, 53).

### Reinfectivity assays and quantitation of chlamydial infectious progeny.

Reinfectivity assays were performed at 24, 36, and 44 or 48 hpi as we described previously (52, 53). Briefly, infected cells in one or two paired shell vials were fixed and stained with Pathfinder (Bio-Rad) as described above; the percent infection was determined. Material in the paired shell vial was serially diluted onto fresh monolayers of the same cell type from the same patient, as in the initial infection, or on fresh HeLa or HCjE cells, and the numbers of IFU per milliliter were calculated at the respective time point.

### DNA extraction and qPCR.

DNA purification and RNase A treatment of cells grown in 24-well plates at 24, 36, and 44 or 48 hpi were performed using the MasterPure kit (Epicentre) per the manufacturer’s protocol. qPCR was performed to calculate sample genome copy number using a standard curve of serial dilutions of a plasmid containing the *ompA* gene of known concentration, as we described previously (52, 53, 114).

### Meso-scale detection (MSD) of cytokines and chemokines.

Supernatants (50:50 KGM-RPMI 1640) were collected from each cell type at 30 and 44 to 48 hpi as indicated in the figures and diluted 1:2 or 1:4 in appropriate buffer in duplicate for analysis of 29 cytokines and chemokines using human cytokine 30-plex V-PLEX array plates, analyzed on a Meso QuickPlex SQ120 instrument (Meso Scale Diagnostics, Rockville, MD) according to the manufacturer’s instructions. Analysis was performed using the manufacturer’s protocol, in which numbers of picograms per milliliter (from 0.02 pg/ml to 3.26 pg/ml, depending on the analyte) were calculated on the basis of standard curves for each analyte, which were also used to verify the manufacturer’s determined lower limit of detection (LLOD). The numbers of picograms per milliliter from uninfected cell supernatants were subtracted from picogram-per-milliliter concentrations of the respective infected cell supernatants for raw data presentation. For fold change calculations, the concentration of each analyte from an infected cell supernatant was divided by the concentration of the analyte in the corresponding uninfected cell supernatant. Duplicates for each cell type from each patient and two to three independent experiments were performed as described above. Follow-up V-PLEX assays were performed as described above using 20 analytes that displayed infection-induced changes from the uninfected cells. When picogram-per-milliliter measurements were below the LLOD for a given analyte, the LLOD was used in fold change calculations.

### Statistical analysis.

Data are expressed as means ± standard deviations (SD) for independent experiments. For multiple comparisons, a one-way analysis of variance (ANOVA) was performed with a Fisher’s least significant difference (LSD) posttest. When comparing two groups, significance was determined using Student's *t* test. For MSD data only, where indicated in the figure legends, fold change was calculated based on comparison with the uninfected control and log_10_ transformed before we performed statistical tests. To determine significant increases in cytokine or chemokine levels, a threshold for fold change was determined based on plotting the log of all fold change data for each analyte (setting the threshold at 2 standard deviations above or below the mean of the log fold change distribution) and set at ±2. A *P* value of <0.05 was considered significant. Prism software was used for statistical analyses.
