# Supplementary material for: Stromal Fibroblasts Drive Host Inflammatory Responses That Are Dependent on Chlamydia trachomatis Strain Type and Likely Influence Disease Outcomes
Source: mBio. 2019 Mar 19;10(2):e00225-19. doi: 10.1128/mBio.00225-19 (PMC6426598; doi:10.1128/mBio.00225-19)
Supplement: TABLE S1 [file mBio.00225-19-st001.pdf]

Table S1. Patient sources of primary cells and cell population characteristics used in designated figures.

Primary cell type	Patient #	Gender, age in years	Cell Composition	Fig 1	Fig 2	Fig 3	Fig 4	Fig 5	Fig 6	Fig 7	Fig S1	Fig S2	Fig S3	Fig S4	Fig S5
CjE	1	M, 42	28% goblet; 69% stratified squamous; 3% fibroblast	X		X	X		X			X	X		
	2	M, 56	85% goblet; 13% stratified; 2% fibroblast	X		X			X			X		X	
	3	M, 60	86% goblet; 4% stratified; 10% fibroblast	X			X		X			X			
	4	M, 61	86% goblet; 14% stratified; 0% fibroblast		X						X				
	5	M, 74	89% goblet; 9% stratified; 2% fibroblast												X
	6	F, 54	7% goblet; 1% stratified; 92% fibroblast	X			X					X			
	7	F, 80	42% goblet; 56% stratified; 2% fibroblast	X		X			X			X			
CjS	1	M, 42	100% fibroblast	X					X				X	X	
	2	M, 56	100% fibroblast	X		X	X					X			
	3	M, 60	100% fibroblast			X						X			
	5	M, 74	100% fibroblast	X	X	X	X		X	X	X	X			
	6	F, 54	100% fibroblast				X					X			
	7	F, 80	100% fibroblast	X					X						X

	8	F, 56	100% fibroblast						X						
EcE	9	F, <50	95% epithelial; 5% fibroblast	X		X			X			X	X		
	10	F, <50	41% epithelial; 59% fibroblast	X											
	11	F, <50	55% epithelial; 45% fibroblast	X			X		X			X			
	12	F, <50	61% epithelial; 39% fibroblast	X			X		X			X			
	13	F, <50	100% epithelial			X						X			X
	14	F, <50	100% epithelial		X	X	X		X		X	X		X	
EcS	9	F, <50	100% fibroblast						X				X		
	10	F, <50	100% fibroblast	X		X	X		X			X			
	12	F, <50	100% fibroblast	X			X	X	X			X			
	13	F, <50	100% fibroblast	X	X	X	X				X	X			X
	14	F, <50	100% fibroblast	X		X	X		X			X		X	
	15	F, <50	100% fibroblast									X			
EmS	10	F, <50	100% fibroblast	X								X			
	12	F, <50	100% fibroblast	X			X	X	X			X			
	13	F, <50	100% fibroblast						X						X
	14	F, <50	100% fibroblast	X	X		X		X		X			X	
	15	F, <50	100% fibroblast			X						X			
	16	F, <50	100% fibroblast	X		X	X					X			
	17	F, <50	100% fibroblast	X		X	X		X			X	X		
	18	F, <50	100% fibroblast							X					
