# Supplementary material for: Stromal Fibroblasts Drive Host Inflammatory Responses That Are Dependent on Chlamydia trachomatis Strain Type and Likely Influence Disease Outcomes
Source: mBio. 2019 Mar 19;10(2):e00225-19. doi: 10.1128/mBio.00225-19 (PMC6426598; doi:10.1128/mBio.00225-19)
Supplement: FIG S1 [file mBio.00225-19-sf001.pdf]

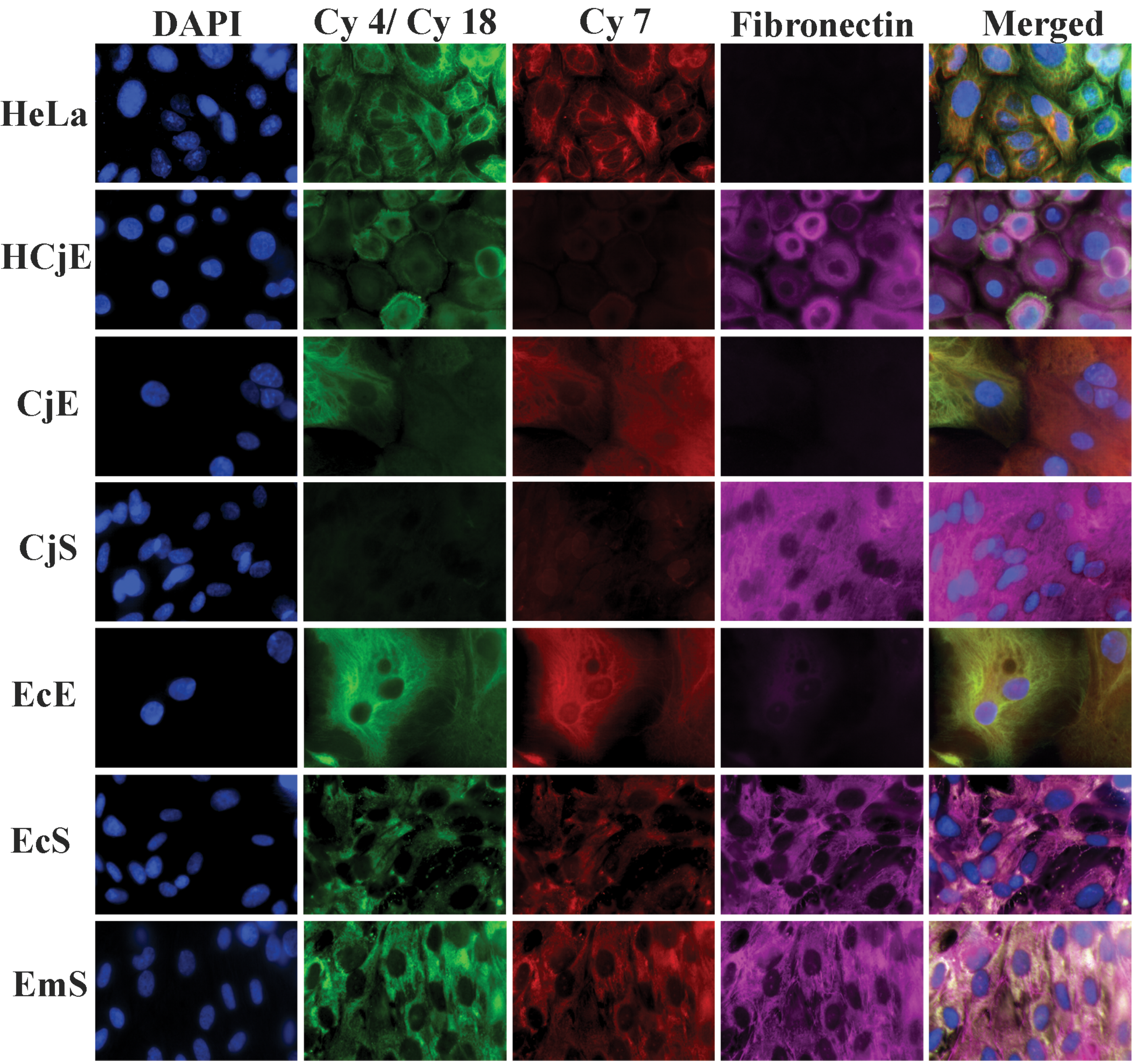

Supplemental Figure 1. Primary cell composition and cell type validation using cytokeratin and fibronectin-specific antibodies. Each cell type was grown to 80% confluence on glass bottom 24 well plates prior to fixing and staining (see MATERIALS AND METHODS). The cells were labeled with squamous epithelial cell-specific cytokeratin 4 antibody (green), goblet cell-specific cytokeratin 7 antibody (red), columnar epithelial cell-specific cytokeratin 18 antibody (green), stromal cell-specific fibronectin antibody (cyan) and with Hoescht (blue) for nuclear and bacterial DNA. Primary conjunctival epithelial (CjE) cell preparations had a range of 40-80% goblet cells and 20-60% squamous cells, depending on the dissection (see MATERIALS AND METHODS). All stromal cells were verified to be free of epithelial cell contamination. Imaging was performed on a Nikon Eclipse Ti-E inverted microscope with an LED illumination system and a DS-Qi2 camera at 90x magnification.
