# Supplementary material for: Stromal Fibroblasts Drive Host Inflammatory Responses That Are Dependent on Chlamydia trachomatis Strain Type and Likely Influence Disease Outcomes
Source: mBio. 2019 Mar 19;10(2):e00225-19. doi: 10.1128/mBio.00225-19 (PMC6426598; doi:10.1128/mBio.00225-19)
Supplement: FIG S2 [file mBio.00225-19-sf002.pdf]

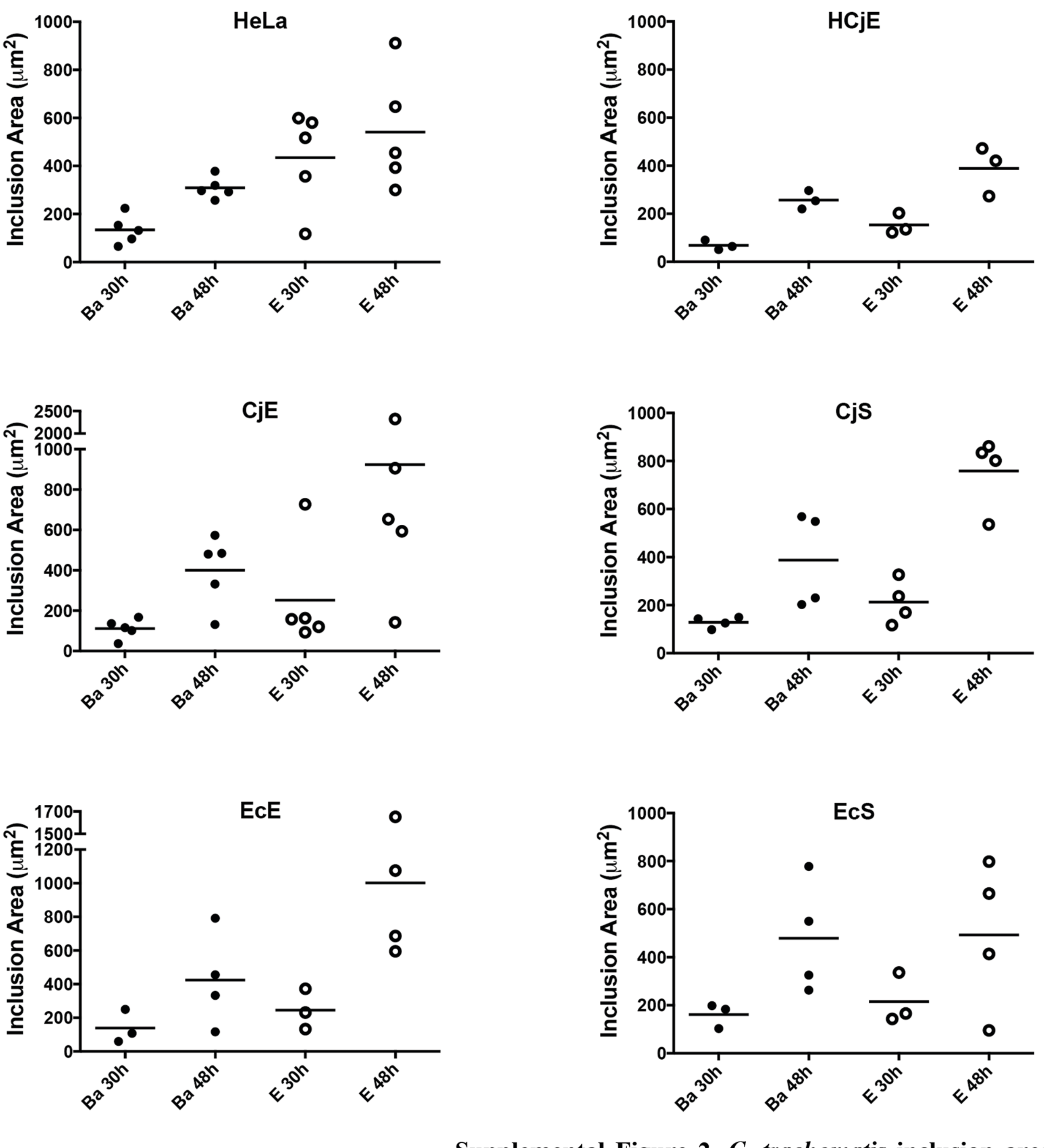

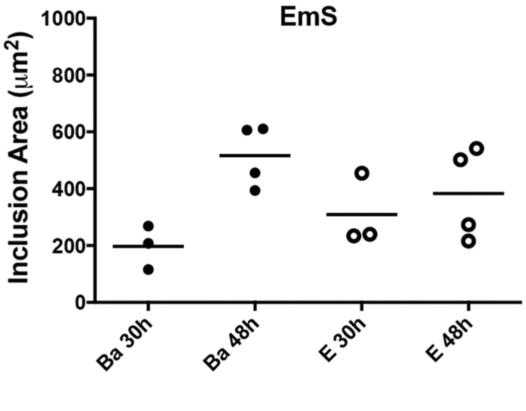

Supplemental Figure 2. *C. trachomatis* inclusion areas at 30 and 48 hpi. HeLa 229 and HCjE cells, and primary CjE, CjS, EcE, EcS and EmS cells were grown on tissue culture treated plastic plates and infected with Ba/Apache-2 or E/Bour at an MOI of 1. The cells were fixed and stained at 30 or 48 hpi (see MATERIALS AND METHODS). For determination of the inclusion areas, images were acquired using 40x1.5x air objective with an NA of 0.6 on a Nikon Eclipse Ti-E inverted microscope. Elements software was used to calculate the inclusion areas expressed as μm^2. Each filled or open circle represents one patient sample for the specified cell type. The horizontal line represents the mean inclusion area.
