# Supplementary material for: Stromal Fibroblasts Drive Host Inflammatory Responses That Are Dependent on Chlamydia trachomatis Strain Type and Likely Influence Disease Outcomes
Source: mBio. 2019 Mar 19;10(2):e00225-19. doi: 10.1128/mBio.00225-19 (PMC6426598; doi:10.1128/mBio.00225-19)
Supplement: FIG S3 [file mBio.00225-19-sf003.pdf]

Figure 3.

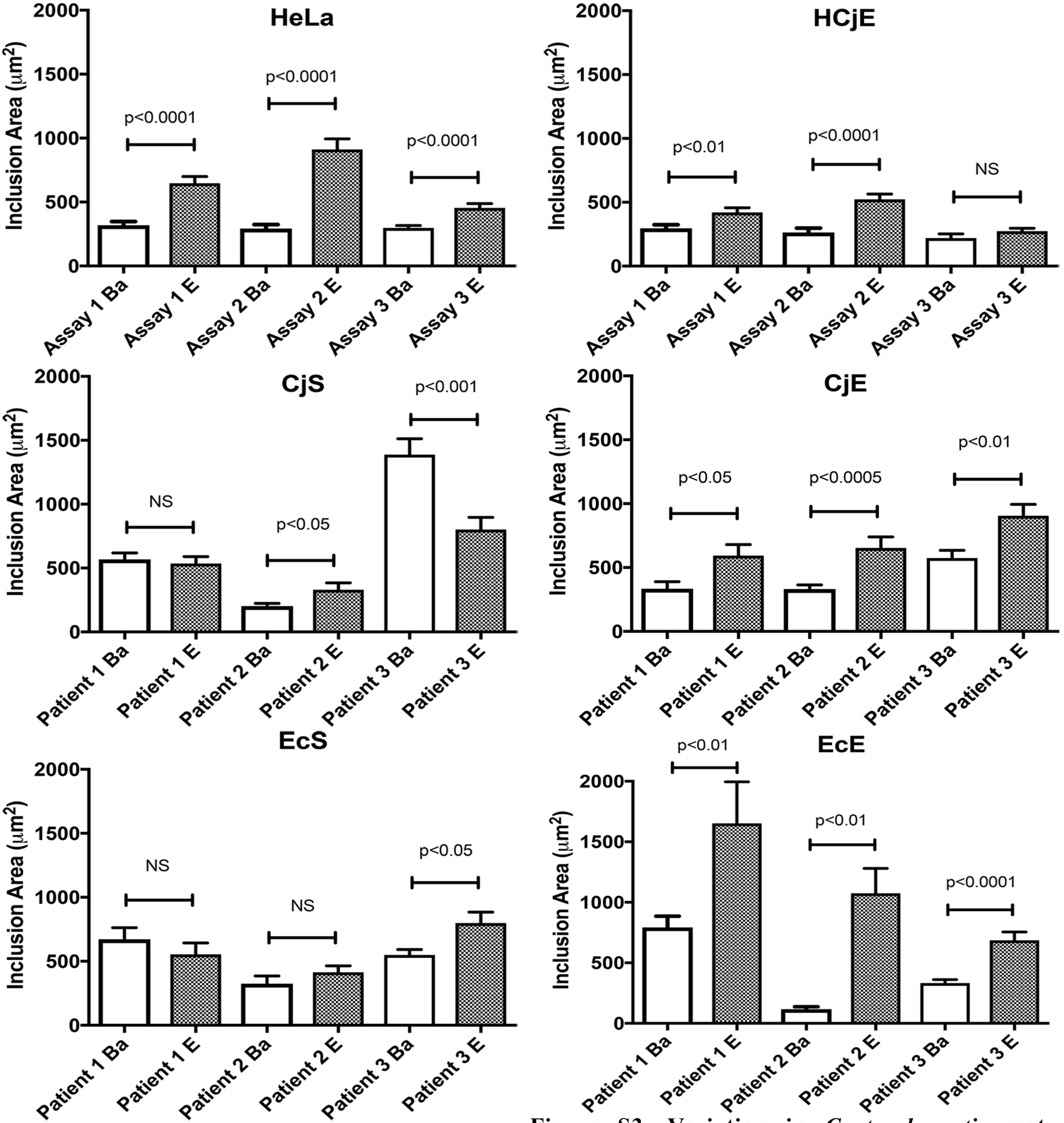

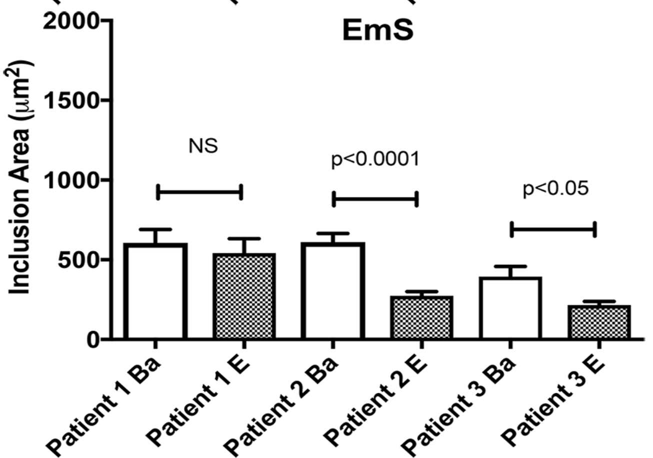

Figure S3. Variation in *C. trachomatis* mature inclusion area is dependent on patient of origin for each primary cell-type. HeLa 229 and HCjE cells, and primary CjE, CjS, EcE, EcS and EmS cells were infected with Ba/Apache-2 or E/Bour at an MOI of 1, and stained at 48 hpi (see MATERIALS and METHODS). For inclusion areas, images were acquired using 40x1.5x air objective with an NA of 0.6 on a Nikon Eclipse Ti-E inverted microscope. Elements software was used to calculate inclusion areas expressed as μm^2. Three independent experiments for immortalized cells and cells from three patients for each cell-type were compared. For CjE and EcE cells, inclusion areas were measured only for epithelial cells and not for any contaminating stromal cells, if present. NS, not significant.
