# Supplementary material for: Stromal Fibroblasts Drive Host Inflammatory Responses That Are Dependent on Chlamydia trachomatis Strain Type and Likely Influence Disease Outcomes
Source: mBio. 2019 Mar 19;10(2):e00225-19. doi: 10.1128/mBio.00225-19 (PMC6426598; doi:10.1128/mBio.00225-19)
Supplement: FIG S4 [file mBio.00225-19-sf004.pdf]

Figure 4.

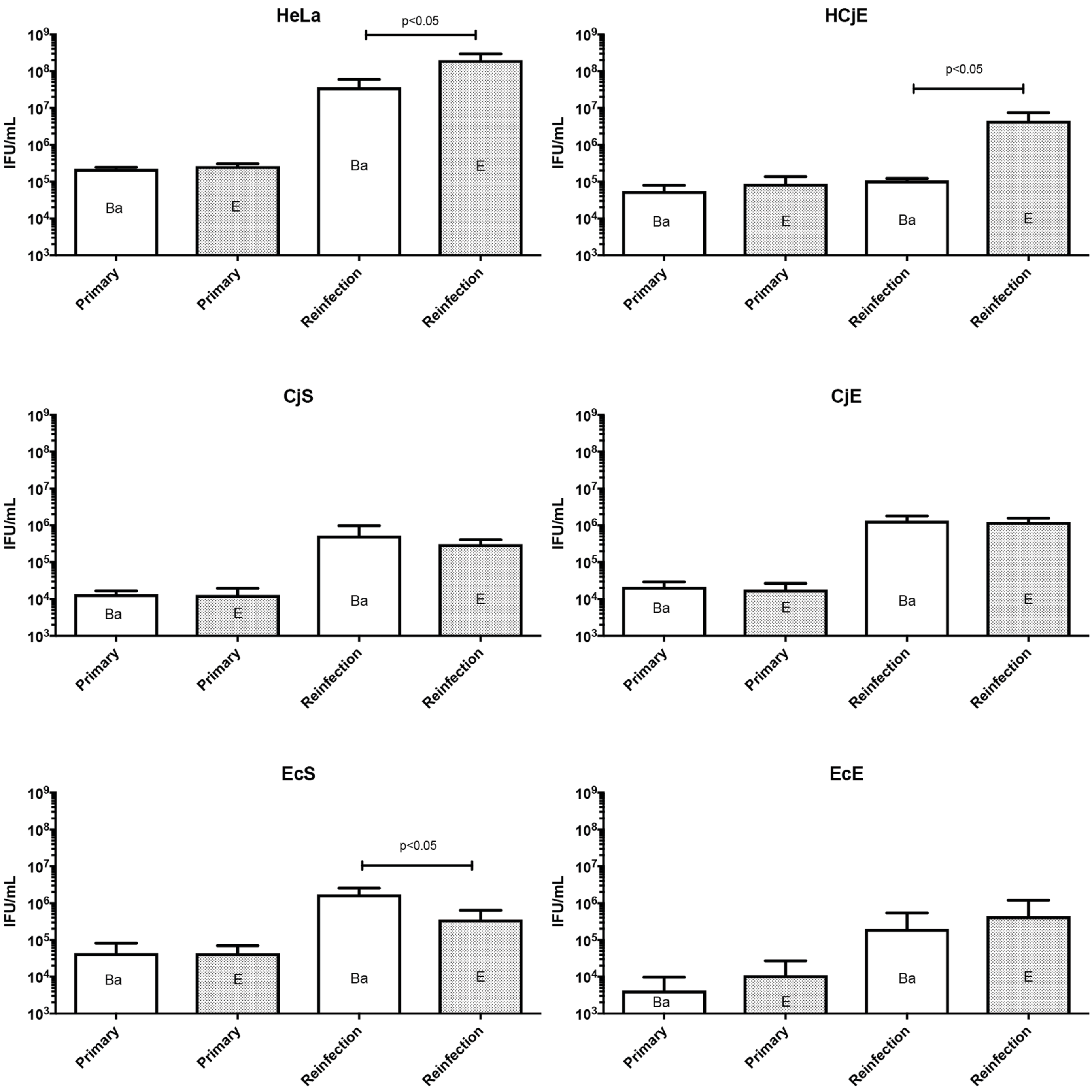

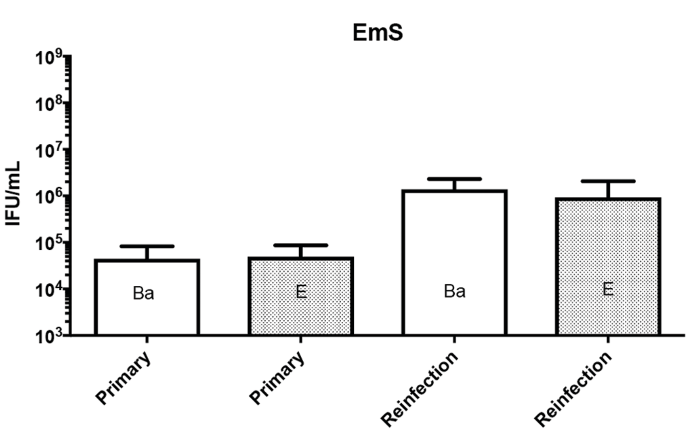

Figure S4. Infectious progeny production by *C. trachomatis* ocular and urogenital strains differs among cell-types. HeLa 229 and HCjE cells, and primary CjE, CjS, EcE, EcS and EmS cells were infected with Ba/Apache-2 or E/Bour at an MOI of 1. At 48 hpi, cultures were serially diluted onto the same cell-types from the same patients as the primary infection. Cells were fixed and stained at 48 hpi, and IFUs/mL was determined (see MATERIALS AND METHODS). The values primary infections represent mean and standard deviation for three to four independent experiments for immortalized cells and for three to six patients per cell-type for primary cells. Values for re-infection represent three to four independent experiments/patients for each cell type.
