# Supplementary material for: Stromal Fibroblasts Drive Host Inflammatory Responses That Are Dependent on Chlamydia trachomatis Strain Type and Likely Influence Disease Outcomes
Source: mBio. 2019 Mar 19;10(2):e00225-19. doi: 10.1128/mBio.00225-19 (PMC6426598; doi:10.1128/mBio.00225-19)
Supplement: TABLE S2 [file mBio.00225-19-st002.pdf]

Supplemental Table 2. Analytes that were up- or down-regulated in response to *Ct* compared to mock-infected primary cells (data taken from **Figure 4**).

**Primary cell type**	**IL-1β**	**IL-2**	**IL-12p70**	**TNF-α**	**GM-CSF**	**IL-1α**	**Eotaxin**	**Eotaxin-3**	**MIP-1α**	**MIP-1β**
CjE	X		X			X	X		X	X
CjS	X	X	X	X	X	X	X	X	X	X
EcE	X	X	X	X	X	X		X	X	
EcS	X	X	X	X	X	X				X
EmS	X	X	X	X	X	X		X	X	X
	**TARC**	**IL-8**	**MCP-1**	**MCP-4**	**IL-4**	**IL-6**	**IL-10**	**IL-13**	**IFN-γ**	**IP-10**
CjE	X	X	X	X						X
CjS	X	X	X	X	X	X	X	X	X	X
EcE		X	X	X	X	X		X		X
EcS		X	X	X	X	X	X	X	X	X
EmS	X	X	X	X	X	X	X	X	X	X
