# Supplementary material for: Stromal Fibroblasts Drive Host Inflammatory Responses That Are Dependent on Chlamydia trachomatis Strain Type and Likely Influence Disease Outcomes
Source: mBio. 2019 Mar 19;10(2):e00225-19. doi: 10.1128/mBio.00225-19 (PMC6426598; doi:10.1128/mBio.00225-19)
Supplement: FIG S5 [file mBio.00225-19-sf005.pdf]

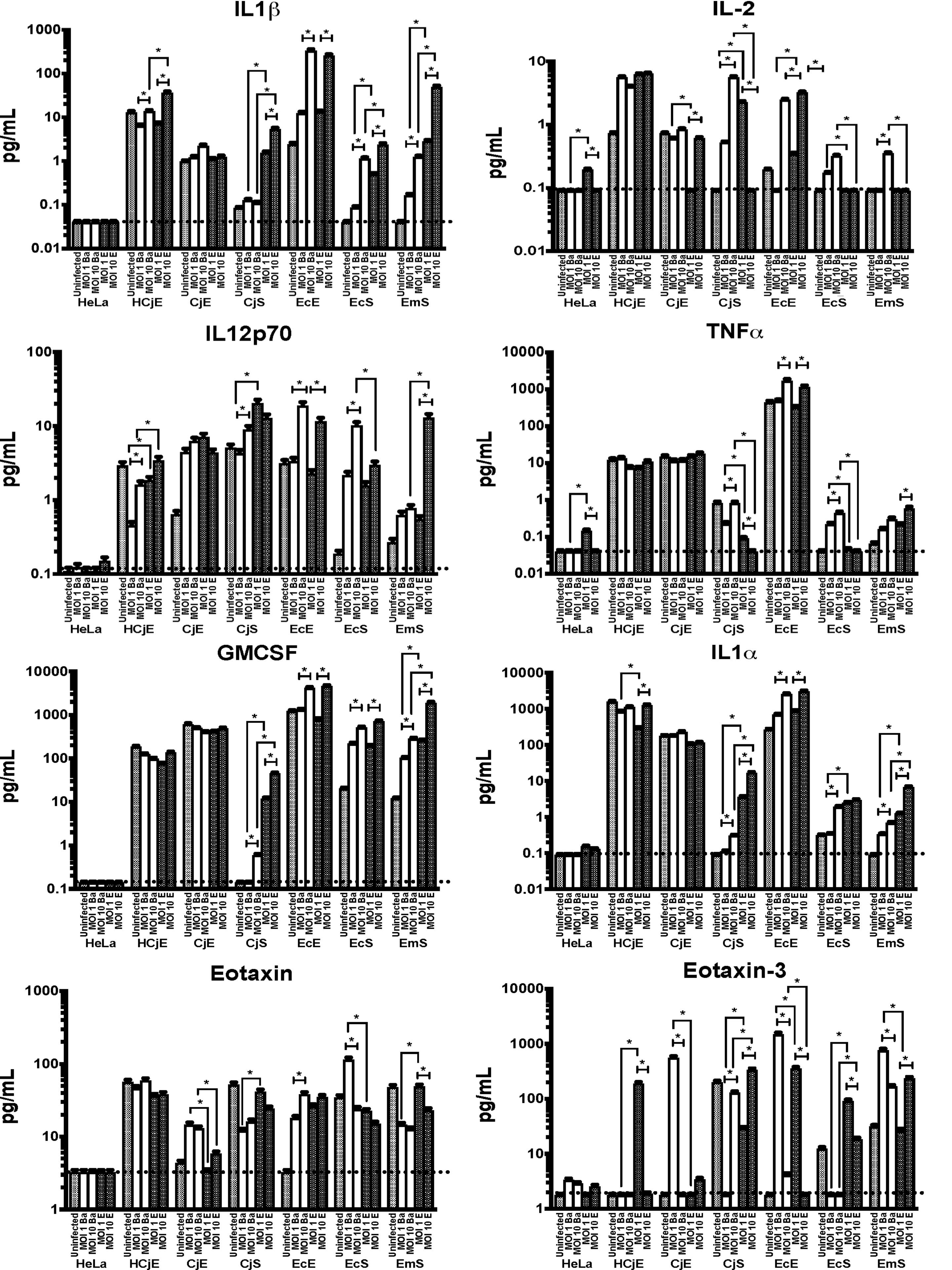
IL1β
IL-2
IL12p70
TNFα
GMCSF
IL1α
Eotaxin
Eotaxin-3
pg/mL
Uninfected
MOI 1 Ba
MOI 10 Ba
MOI 1 E
MOI 10 E
HeLa
HCjE
CjE
CjS
EcE
EcS
EmS
0.01
0.1
1
10
100
1000
10000
*

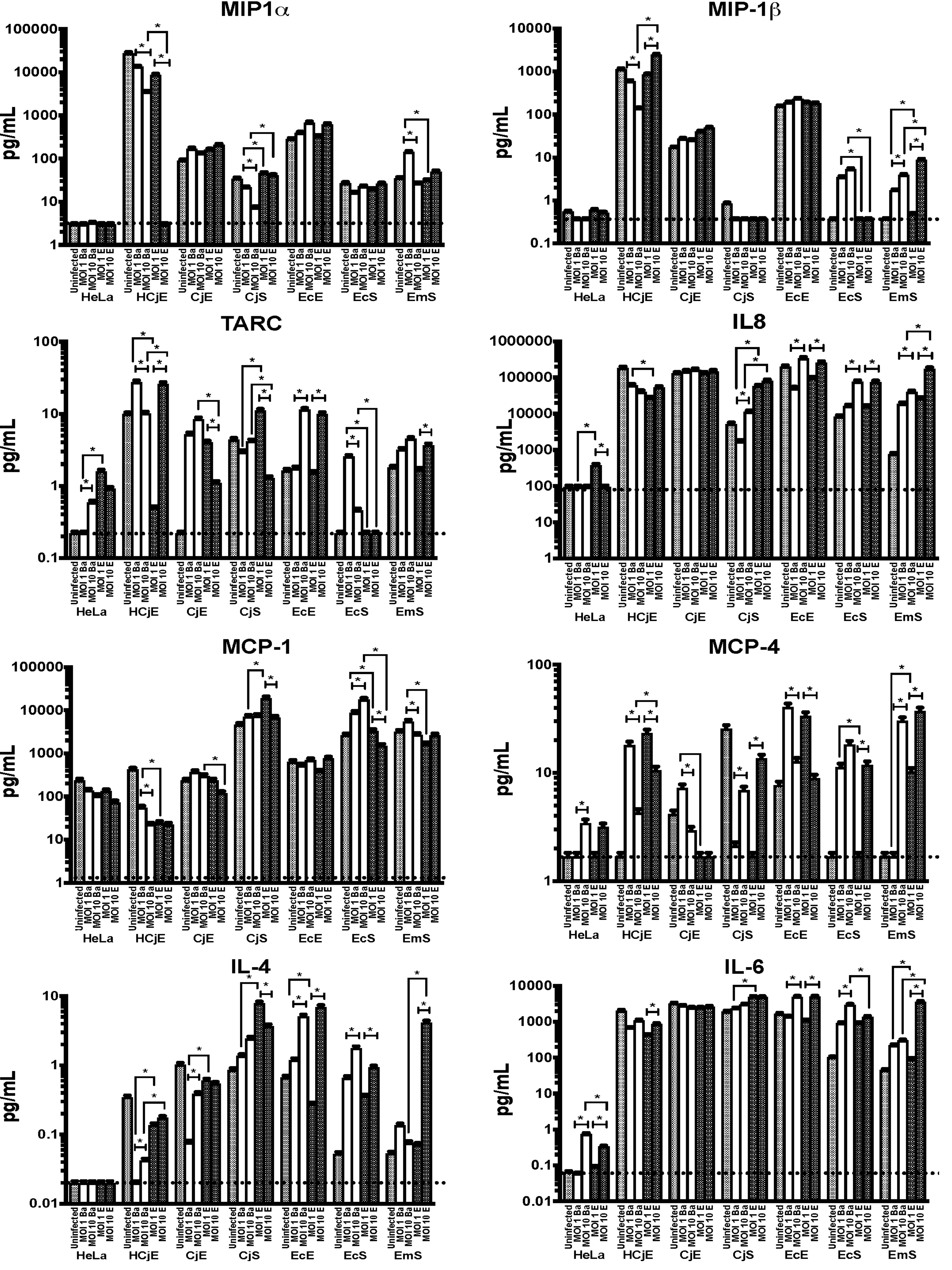

MIP1α
MIP-1β
TARC
IL8
MCP-1
MCP-4
IL-4
IL-6
pg/mL
Uninfected
MOI 1 Ba
MOI 10 Ba
MOI 1 E
MOI 10 E
HeLa
HCjE
CjE
CjS
EcE
EcS
EmS

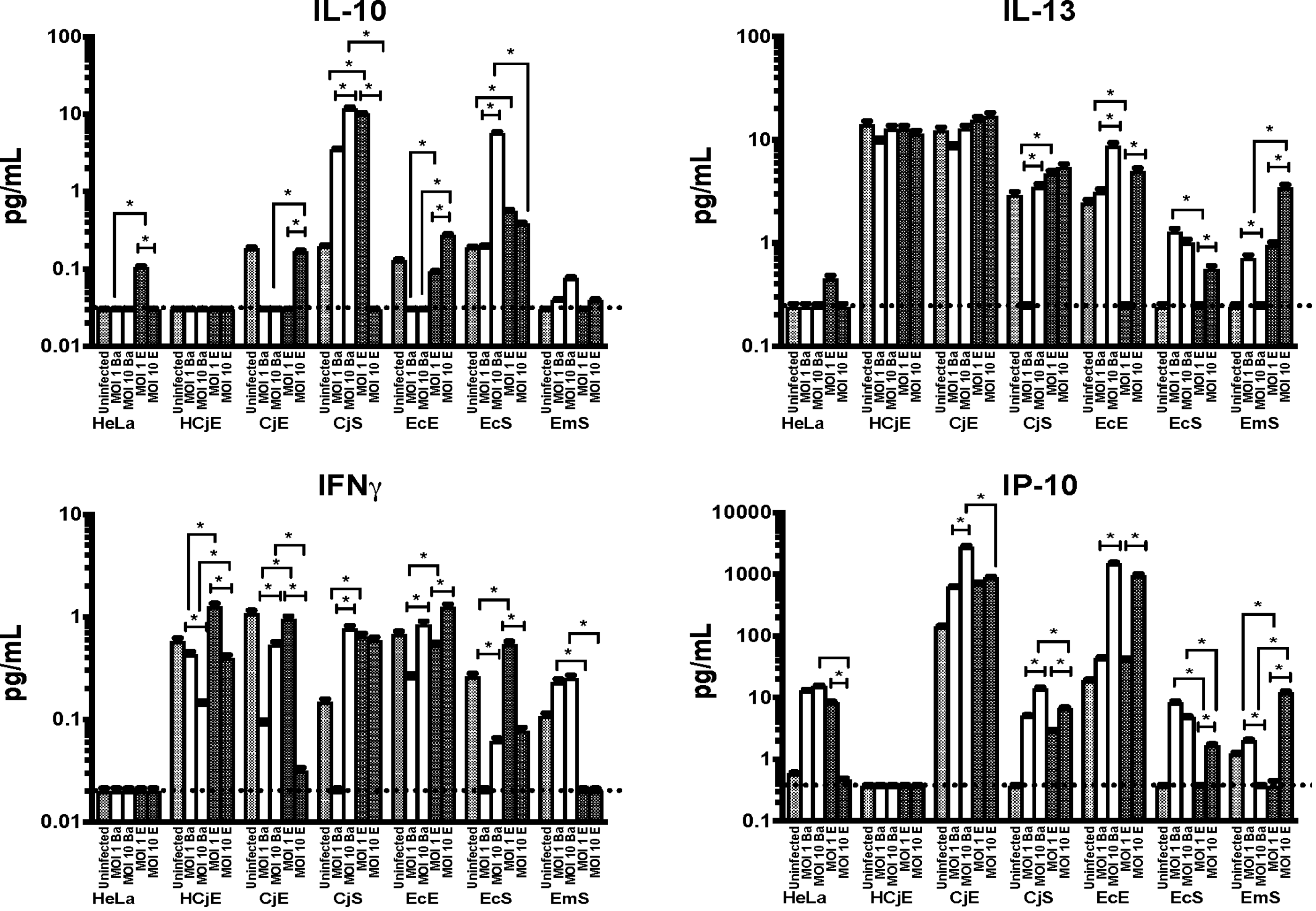

Supplemental Figure 5. Cytokine and chemokine secretion varies depending on the multiplicity of infection (MOI) for *C. trachomatis* ocular and urogenital strains. Immortalized HeLa229 and HCjE cells, and primary CjE, CjS, EcE, EcS and EmS cells were infected with Ba/Apache-2 or E/Bour at an MOI of 1 or 10 or mock infected. The supernatants were collected at 48 hpi and analyzed using the Meso Scale Discovery (MSD) human cytokine/chemokine V-PLEX arrays for 20 analytes (see MATERIALS AND METHODS). The dotted line indicates the lower limit of detection (LLOD) for that analyte, based on the standard curve. To determine whether there was a significant increase in secretion between an MOI of 1 and an MOI of 10, a threshold for fold change was set at ± 2; *, indicates a greater than 2-fold increase (see MATERIALS AND METHODS). The data reflect three independent experiments for immortalized cells and for CjE and CjS cells from a representative male patient, and EcE, EcS, and EmS cells from a representative female patient.
